# Supplementary material for: Causal phase-dependent control of non-spatial attention in human prefrontal cortex
Source: Nat Hum Behav. 2024 Feb 16;8(4):743–57. doi: 10.1038/s41562-024-01820-z (PMC11045450; doi:10.1038/s41562-024-01820-z)
Supplement: Supplementary file 1 — Supplementary Figs. 1–11 and Tables 1–6. [file 41562_2024_1820_MOESM1_ESM.pdf]

nature human behaviour

Article

https://doi.org/10.1038/s41562-024-01820-z

Causal phase-dependent control of non-spatial attention in human prefrontal cortex

In the format provided by the authors and unedited

Table of contents

Supplementary Figures:

Supplementary Figure 1: Control "no-attention" task in Experiments 1 and 2 2

Supplementary Figure 2: The coordinates of the IFJ found in our fMRI experiment match the location of the IFJ in the literature well 2

Supplementary Figure 3: Wide activations of the visual cortex 3

Supplementary Figure 4: dWPLI over the frequency space 3

Supplementary Figure 5: Overlay of fMRI activations and EEG source analysis results 4

Supplementary Figure 6: Microsaccade analysis shows no significant differences between motion, scene or no-attention trials, neither between in-phase or out-of-phase trials 5

Supplementary Figure 7: tACS electric field predictions and coregistration procedure 6

Supplementary Figure 8: Visual stimulus and transcranial electrical stimulation timing 7

Supplementary Figure 9: Performance is stable over time 8

Supplementary Figure 10: The tACS-induced behavioral changes in sensory discrimination of the cued feature are not likely to be influenced by behaviorally-induced effects in the irrelevant feature 9

Supplementary Figure 11: The preferred timing of the electrical stimulation is slightly before the peak activation of the IFJ 10

Supplementary Tables:

Supplementary Table 1: fMRI clusters: Attention > No-attention 11

Supplementary Table 2: fMRI clusters: Motion > No-attention 11

Supplementary Table 3: fMRI clusters: Scene > No-attention 11

Supplementary Table 4: fMRI clusters: Motion > Scene 12

Supplementary Table 5: fMRI clusters: Scene > Motion 12

Supplementary Table 6: EEG clusters: Attention > No-attention 12

References 12

Supplementary Fig. 1

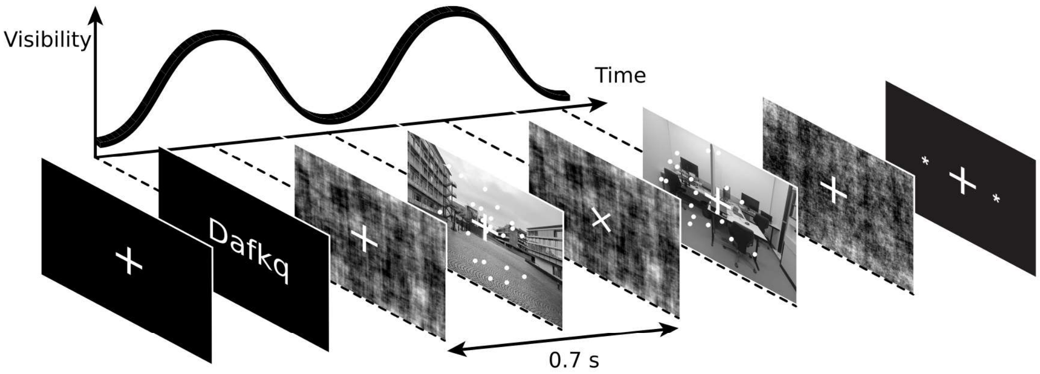

Control "no-attention" task in Experiments 1 and 2. To study brain activity purely evoked by the visual input, without the influence of attention, in the fMRI and EEG experiments participants first carried out a version of the task without non-spatial attention. They were instructed to pay attention to the fixation cross and to press a button when the fixation cross made a 45° orientation shift. These orientation shifts would happen at random intervals, uniformly distributed between 5 and 30 seconds. The visual information on the screen was identical to the non-spatial attention task, with the exception that all text was replaced by nonsense text and the fixation cross was visible at all times.

Supplementary Fig. 2

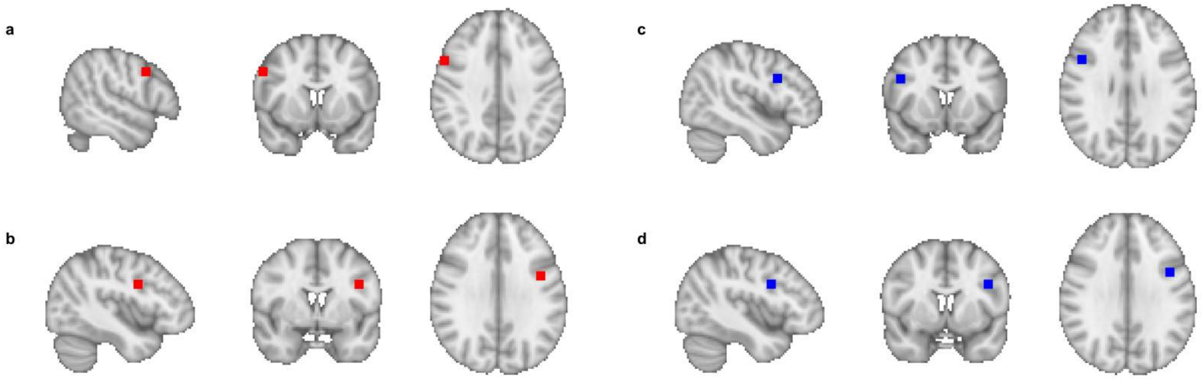

The coordinates of the IFJ found in our fMRI experiment match the location of the IFJ in the literature well. a We find a peak activation (highest Z-score) at MNI = 54, 10, 36, for the right IFJ. **b** For the left IFJ we find a peak activation at MNI = -42, 2, 30. **c** Bedini et al (1) used activation likelihood estimations across a large set of fMRI localizer studies to find the right IFJ at MNI = 46, 12, 28. **d** The left IFJ was found at MNI = -42, 6, 30.

Supplementary Fig. 3

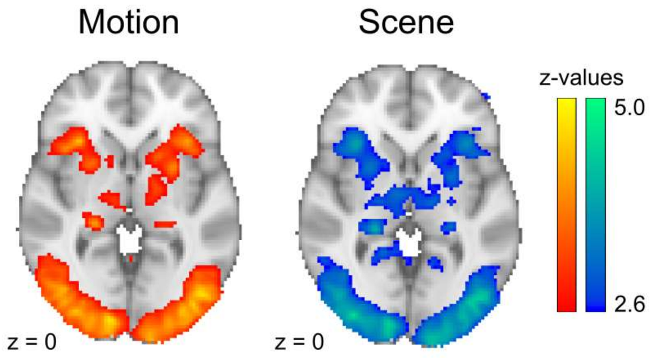

Wide activations of the visual cortex. Using fMRI without contrasting attentive vs. no-attention states we find a wide activation of the visual cortex, both when participants are cued for motion and for scenes. There is a high degree of overlap when participants were cued each sensory modality

Supplementary Fig. 4

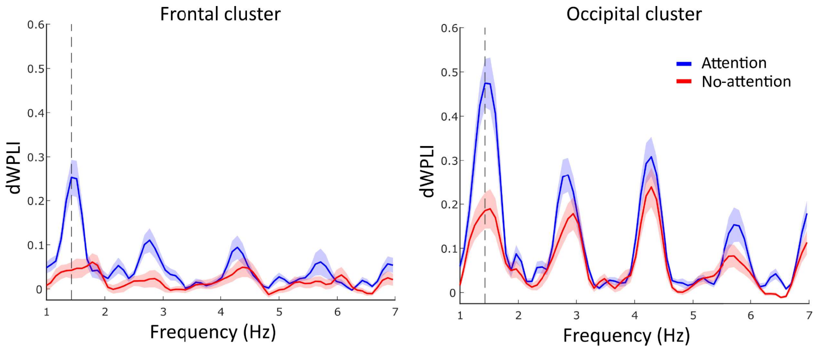

dWPLI over the frequency space. dWPLI values for different frequencies between the sensor data and the visual stimulation signal for the frontal and occipital cluster of electrodes (see Fig. 1e) for the attention (blue) and no-attention (red) tasks. The peaks correspond to the frequency of the visual stimulation signal (1.43 Hz, represented by the dashed line) and its harmonics. Shaded areas represent one standard error of the mean.

Supplementary Fig. 5

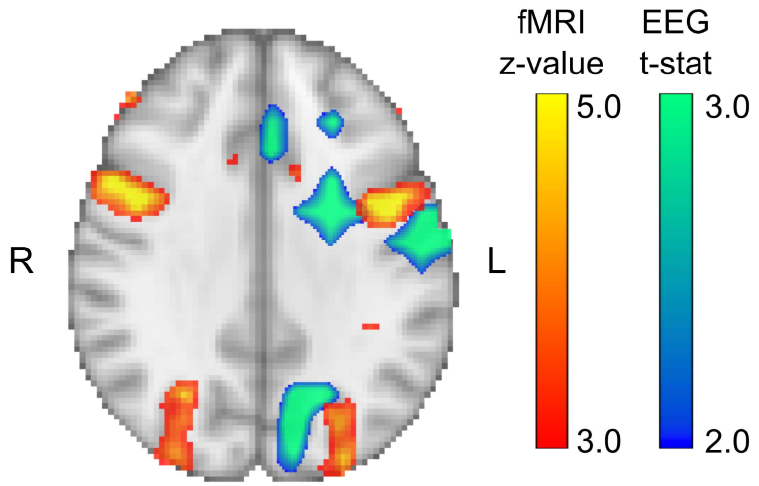

Overlay of fMRI activations and EEG source analysis results. In a contrast of attentive versus no-attention states we find that both the left and right IFJ activate in the fMRI experiment, in the EEG source analysis the results are lateralized towards the left IFJ. The EEG cluster is in the vicinity of the IFJ, but does not exactly overlap, likely due to the lower spatial resolution of EEG.

Supplementary Fig. 6

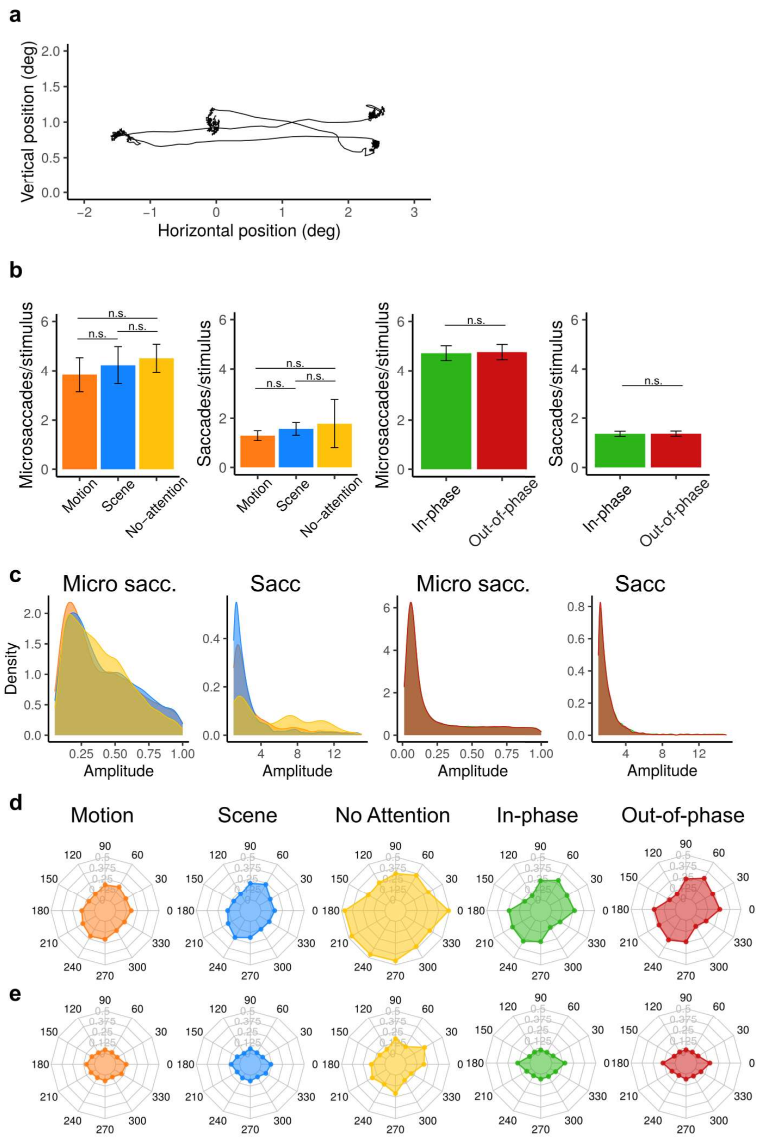

Microsaccade analysis shows no significant differences between motion, scene or no-attention trials, neither between in-phase or out-of-phase trials. **a** Representative example of the gaze position during one trial. The example shows 3 saccades and several microsaccades. **b** Analysis of the eye tracking data shows no significant differences in the number of saccades and microsaccades per stimulus presentation in which motion or scenes were cued, or in no-attention states. There are also no significant differences in saccades per stimulus for in-phase or out-of-phase stimulated trials (All pairwise combinations of two-sided paired t-tests $P > 0.4$). The colors in this panel and the rest of the figure represent the dataset of which the gaze data was taken from: orange, blue, yellow, green and red indicate motion, scene, no-attention, in-phase and out-of-phase, respectively. Motion, scene and no-attention tested n = 20 participants, for in-phase and out-of-phase n = 37. **c** The density of amplitudes show that the imaging results (fMRI and EEG) cannot be explained by differences in eye-movements. We found higher activity in the IFJ in the attentive compared to the non attentive condition, , while amplitudes of eye movements are larger in the non-attentive condition (mean $\pm$ SEM amplitude motion microsaccades = 0.42 $\pm$ 0.01, image microsaccades = 0.42 $\pm$ 0.01, no attention microsaccades = 0.41 $\pm$ 0.01, motion saccades = 3.0 $\pm$ 0.1, image saccades = 2.8 $\pm$ 0.1, no attention saccades = 3.1 $\pm$ 0.1). In the right two panels, the distributions are colored brown since the red and green distributions completely overlap (mean $\pm$ SEM amplitude in-phase microsaccades = 0.24 $\pm$ 0.002, out-phase microsaccades = 0.24 $\pm$ 0.002, in-phase saccades = 2.5 $\pm$ 0.03, out-phase saccades = 2.5 $\pm$ 0.03). **d** The spider plots show that the direction of the microsaccades for the different conditions are similar. **e** Same as d, but for saccades > 1 degree.

Supplementary Fig. 7

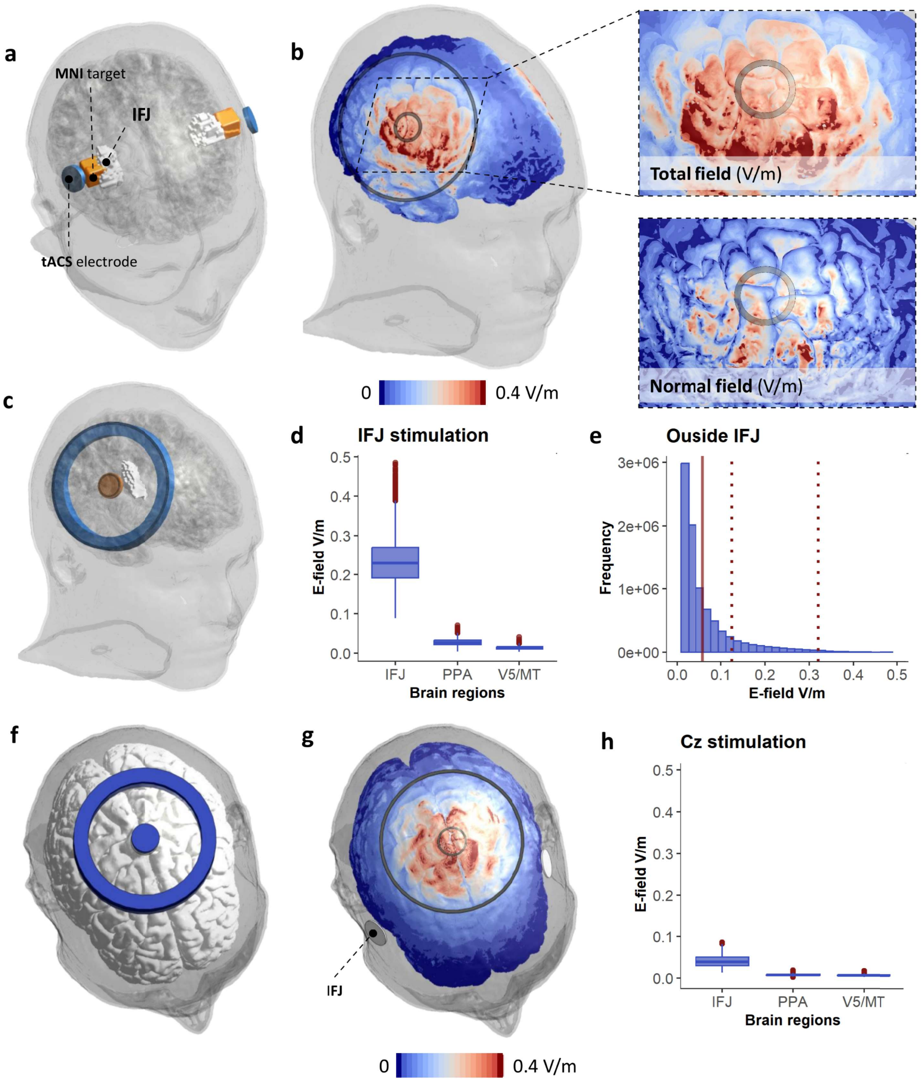

tACS electric field predictions and coregistration procedure. a Model geometry with the identified IFJ (in white) and the stimulation electrodes (round in yellow and ring in blue) above the target. The contour of the electrodes is highlighted in black. The identification of the IFJ in the MIDA was done by coregistering the MIDA with the Brainnetome atlas. **b** The IFJ segmented from the atlas (in white) and target electrode (in blue) displayed together with the cortical target (in yellow) identified in the MNI space. **c** Left: absolute E-field distribution on the cortical surface for an input current of 4 mA (peak-to-peak). Right: Surface views of the total E-field magnitude (top view) and of the normal E-field component to the cortex (bottom view) which is considered to be the principally relevant E-field component coupling with the electrophysiology of pyramidal neurons. **d** Boxplots represent the distribution of the E-field in each voxel within the IFJ (0.24 ± 0.06), PPA (0.028 ± 0.008), and MT+ (0.013 ± 0.004). These results indicate that the relevant sensory areas in this study were not affected by the application of our tACS protocol. Moreover, the fact that influences of our tACS protocol on non-spatial attention were larger in motion relative to scenes cannot be explained by differences in E-field strength as these are negligible in both sensory areas. The line within the boxplot presents the median with the box indicating the 25th percentile (Q1) and the 75th percentile (Q3) the whiskers start at Q1 - 1.5 ·IQR and Q3 + 1.5·IQR with IQR the interquartile range (from Q1 to Q3), dots represent potential outliers. **e** In the control experiment (Experiment 5) the electrodes are placed on the Cz location of the 10-20 EEG coordinate system, therefore stimulating the motor cortex. **f** Same as in d but for the electrical fields as produced in the control experiment. The distributions of the E-field is within the IFJ (0.04 ± 0.01), PPA (0.008 ± 0.002), MT+ (0.007 ± 0.002). **g** Absolute E-field distribution on the cortical surface for an input current of 4 mA on Cz electrode. **h** Same as in d but for the electrical fields as produced in the control experiment. The distributions of the E-field is shown within the IFJ (0.04 ± 0.01), PPA (0.008 ± 0.002), and MT+ (0.007 ± 0.002).

Supplementary Fig. 8

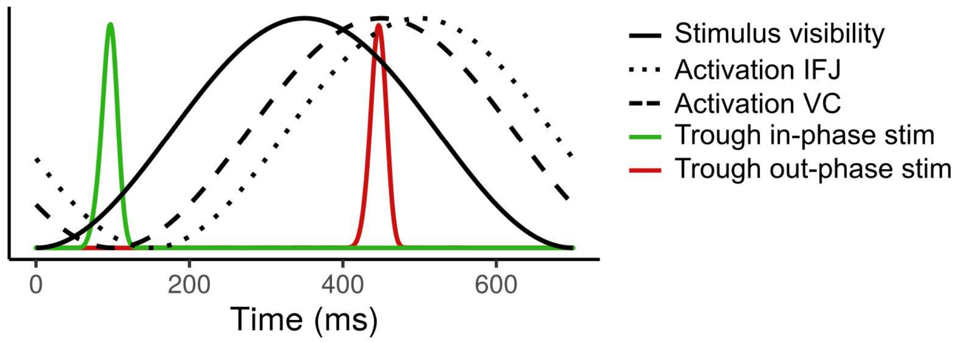

Visual stimulus and transcranial electrical stimulation timing. Based on the results obtained in our EEG experiment, we found that the visual cortex (VC) and the IFJ get entrained to the visual stimulation. The delay between visual stimulation and the response in the visual cortex was about 100 ms and 150 ms for the IFJ. Using photosensitive triggers on the monitor we could record the exact timing of the visual stimulation and compare it to the ongoing electrical stimulation. The timing of the trough of in-phase stimulation (mean = 95 ms after the trough of visual stimulation, SD = 9) is represented in green and out-of-phase (mean = 445 ms after the trough of visual stimulation, SD = 15) in red. By timing the tACS waveform roughly 50 ms before the tagged slow rhythmic fluctuations in visual and prefrontal areas, we hypothesized that we could maximize the influence of the stimulation on behavior.

Supplementary Fig. 9

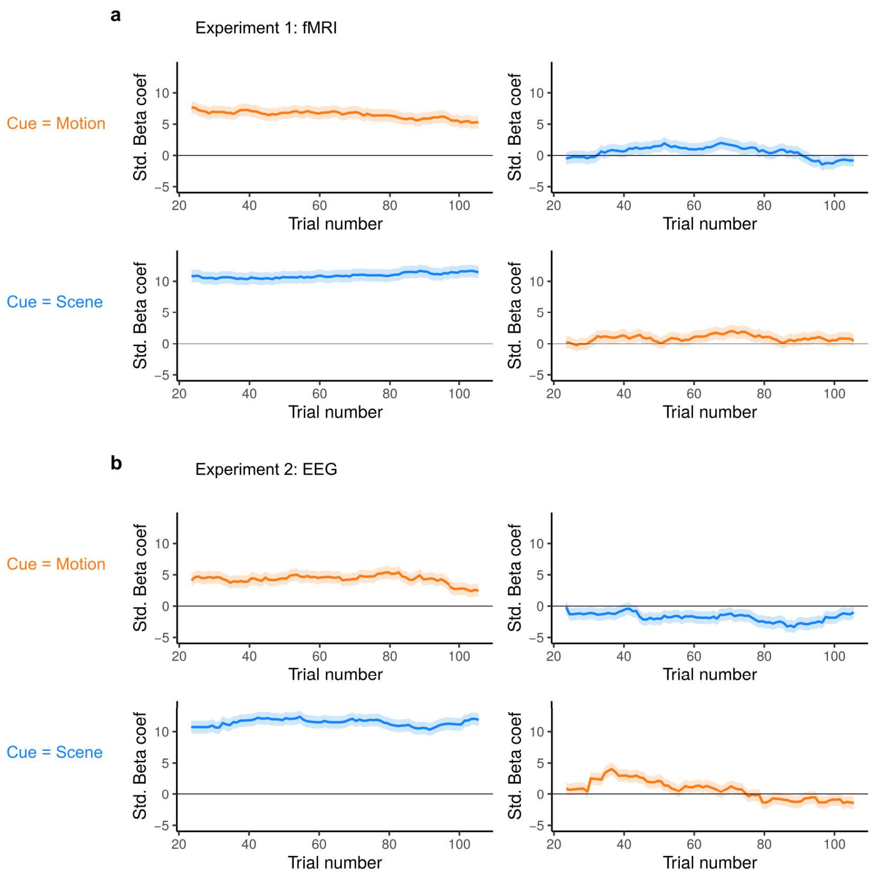

Performance is stable over time. We show naturally occurring fluctuations in performance in time as the main effect of sensory evidence on choice in a linear mixed effects model: Choice $\sim 1+$Scene_evidence$+$Motion_evidence$+(1|$Subject_number$)$. Note the difference with Fig. 2g in which the interaction effect between sensory evidence and stimulation condition was plotted and we observe a clear effect of the stimulation. The grey shaded area indicates the windows for which stimulation was turned on. Lines indicate the expected value, shaded areas around the lines indicate $\pm$ 1 SD of the posterior estimate. **a** Experiment 1: fMRI (n = 20). **b** Experiment 2: EEG (n = 19).

Supplementary Fig. 10

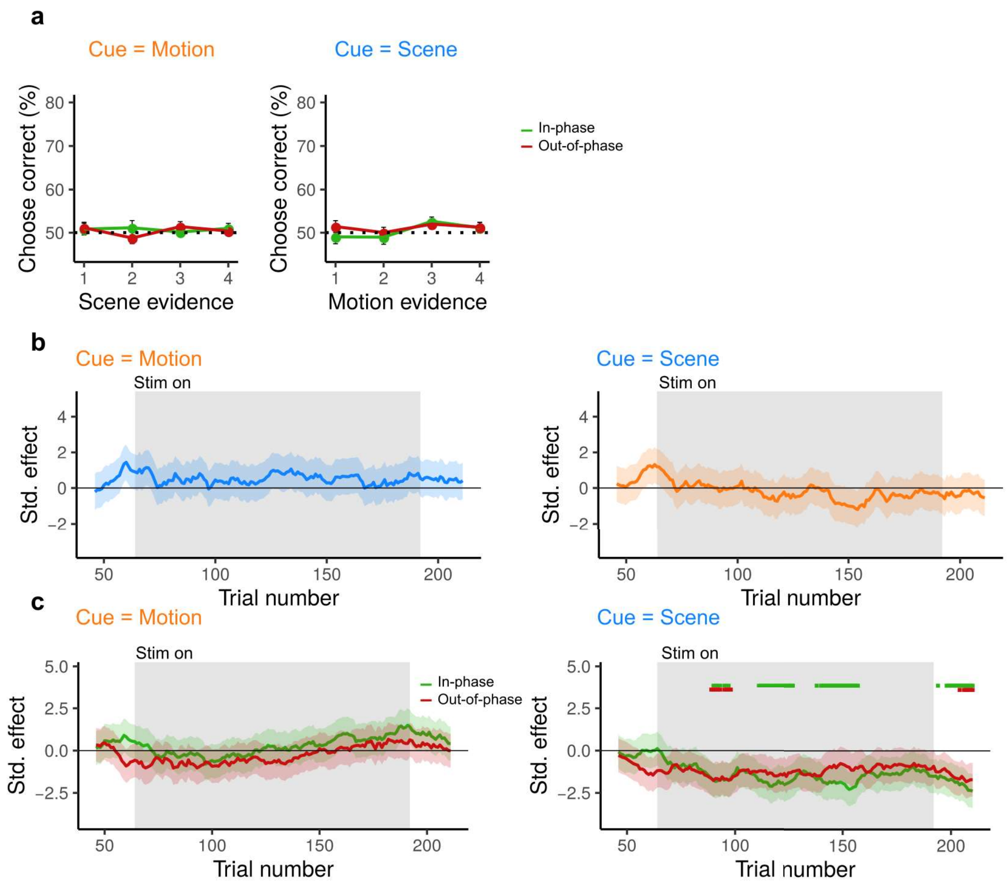

The tACS-induced behavioral changes in sensory discrimination of the cued feature are not likely to be influenced by behaviorally-induced effects in the irrelevant feature (n = 37). a Participants are not distracted by uncued evidence irrespective of the stimulation phase. When participants are cued to motion, scene evidence does not influence their decisions and when cued to scenes motion evidence does not influence their decisions. **b** A moving window analysis shows that there is no significant effect of the stimulation on distraction through time. The grey shaded area indicates the windows for which stimulation was turned on. Lines indicate the expected value, shaded areas around the lines indicate $\pm$ 1 SD of the posterior estimate of the interaction evidence*stimulation. **c** Comparing in-phase and out-of-phase stimulation against baseline performance we find that for trials in which motion is cued neither in-phase nor out-of-phase distraction levels differ from baseline ($P < 0.05$ uncorrected). For scene trials at certain time point participants are significantly distracted by motion evidence. The negative sign of this effect indicates that participants tend to choose outdoor (right button) when motion evidence is towards the left and indoor (left button) when motion evidence is towards the right. Lines indicate the expected value, shaded areas around the lines indicate $\pm$ 1 SD of the posterior estimate of the interaction evidence*stimulation.

Supplementary Fig. 11

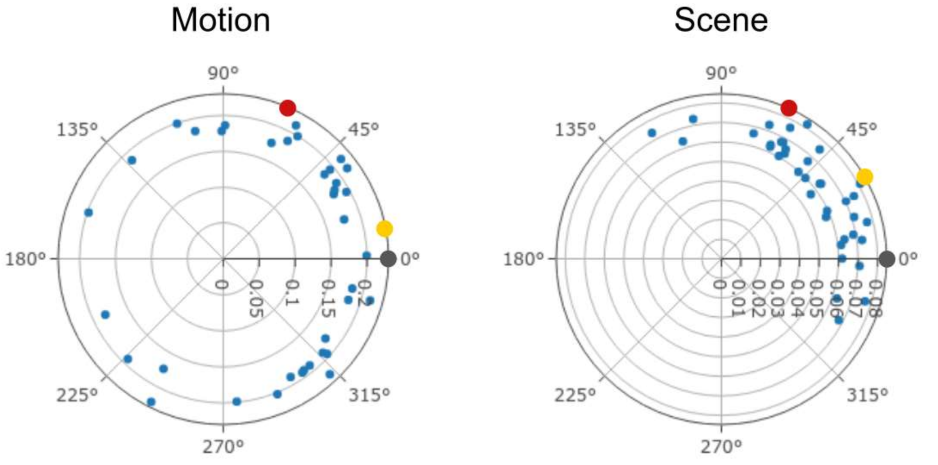

The preferred timing of the electrical stimulation is slightly before the peak activation of the IFJ. Here we plot the preferred phase delay between the visual and electrical stimulation per participant in blue. This is estimated by determining the peak of the sinusoidal function of performance versus stimulation delay in Experiment 4. At 0° the visual stimulation peaks (grey) in yellow the circular mean of the preferred stimulation delay (timing of the peak of the electrical stimulation) is indicated at 7° for motion and 39° for scenes. The peak activation of the IFJ (150 ms later than the visual stimulation) is indicated in red. We see that, in line with experiment 3, the preferred timing of the stimulation is slightly before the peak activation of the IFJ.

Below we show tables of the main fMRI clusters (clusters larger than 100 voxels) for the different contrasts. For table 1-3 Z-statistic images were thresholded at $Z > 4.0$ and a cluster correction was applied at a threshold of $P < 0.05$, for table 4-5 $Z > 2.6, P < 0.05$. The clusters are listed in order of largest cluster size, the location is given in MNI coordinates and the naming is taken from the automated anatomical labelling atlas (AAL3v1) (2).

Table 1. Attention > No-attention

Cluster	Name	LH/RH	Location	Z-value	Voxels
1	Occipital Mid	R	38, -82, 6	7.0	11675
2	Parietal Sup	L	-22, -72, 56	5.7	558
3	Supp Motor Area	-	0, 4, 52	5.5	408
4	Parietal Sup	R	26, -66, 56	6.4	390
5	Precentral (IFJ)	R	54, 10, 36	5.8	387
6	Precentral	L	-38, -6, 62	4.9	343
7	Insula	R	34, 22, 2	5.7	269
8	Insula	L	-36, 24, 2	5.5	215
9	Precentral (IFJ)	L	-42, 2, 30	5.8	196
10	Cerebellum Crus2	-	2, -82, -32	4.8	170
11	Putamen	L	-22, 4, 8	4.7	121

Table 2. Motion > No-attention

Cluster	Name	LH/RH	Location	Z-value	Voxels
1	Cerebellum Crus1	L	-42, -56, -36	5.6	2382
2	Parietal Sup	L	-22, -68, 52	6.2	703
3	Postcentral	L	-48, -8, 50	5.3	381
4	Precentral (IFJ)	R	54, 12, 34	5.9	352
5	Supp Motor Area	-	0, 4, 52	5.6	289
6	Insula	R	32, 20, 2	6.0	270
7	Parietal Sup	R	28, -66, 56	6.5	268
8	Insula	L	-36, 22, 2	5.9	209
9	Precentral (IFJ)	L	-42, 2, 30	5.0	161
10	SupraMarginal	R	44, -36, 44	4.7	140
11	Cerebellum Crus2	-	6, -80, -40	5.1	135

Table 3. Scene > No-attention

Cluster	Name	LH/RH	Location	Z-value	Voxels
1	Cerebellum Crus1	L	-38, -72, -28	5.9	1632
2	Temporal Inf	R	50, -62, -22	5.8	1317
3	Occipital Mid	L	-32, -92, 20	5.6	920
4	Supp Motor Area	-	-2, 4, 54	5.5	555
5	Cerebellum Crus1	-	-10, -80, -24	5.7	439
6	Parietal Sup	R	28, -66, 54	6.1	363
7	Precentral (IFJ)	R	54, 10, 38	5.4	344
8	Insula	R	34, 22, 2	4.8	293
9	Precentral	L	-28, -2, 58	5.7	220
10	Insula	L	-32, 16, 10	5.6	188
11	Precentral (IFJ)	L	-40, 4, 30	5.6	171

Table 4. Motion > Scene

Cluster	Name	LH/RH	Location	Z-value	Voxels
1	Parietal Inf	R	38, -42, 50	3.8	424
2	Parietal Sup	L	-34, -46, 56	3.9	330
3	Occipital Mid (MT+)	L	-44, -76, 2	4.1	315

Table 5. Scene > Motion

Cluster	Name	LH/RH	Location	Z-value	Voxels
1	Fusiform (PPA)	R	26, -46, -16	5.5	3364
2	Fusiform (PPA)	L	-28, -46, -12	5.4	2333
3	Occipital Mid	L	-32, -68, 38	4.5	1403
4	Calcarine	L	-14, -60, 12	4.3	1052
5	Frontal Inf Tri	L	-54, 28, 22	4.3	327
6	Cingulate Mid	-	0, -38, 36	3.9	259

The table below shows the EEG clusters (cluster correction of $P < 0.01$) at the source level for the difference in dWPLI between the attention and no-attention tasks with a significance level of $P < 0.05$. The clusters are listed in order of largest cluster size, the location is given in MNI coordinates and the naming is taken from the automated anatomical labelling atlas (AAL3v1) (2). All spatial neuroimaging claims in this work are based on the fMRI results. Nonetheless, our work provides an opportunity to compare EEG source analysis based on frequency tagging and fMRI results. With the exception of the right Insula, the locations found are different, but in similar brain regions. These differences are likely due to the weaker spatial resolution of EEG. Cluster 4 is the cluster near the IFJ that we refer to as IFJ cluster. Notice that the voxel size in the EEG source analysis is $1\,cm^3$, which explains why the clusters contain fewer voxels compared to the fMRI results.

Table 6. Attention > No-attention

Cluster	Name	LH/RH	Location	t-statistic	Voxels
1	OFC Post	L	-24, 17, -16	4.59	75
2	Cerebellum Vermis 3	-	-1, -37, -13	4.21	36
3	Cuneus	-	0, -85, 40	4.47	34
4	Postcentral	L	-56, -10, 29	4.14	13
5	Insula	R	30, 26, 8	3.54	12

References

1. Marco Bedini, Emanuele Olivetti, Paolo Avesani, and Daniel Baldauf. Accurate localization and coactivation profiles of the frontal eye field and inferior frontal junction: an ALE and MACM fMRI meta-analysis. *Brain Structure and Function*, 228(3):997–1017, 2023. ISSN 18632661. doi: 10.1007/s00429-023-02641-y.
2. Edmund T. Rolls, Chu Chung Huang, Ching Po Lin, Jianfeng Feng, and Marc Joliot. Automated anatomical labelling atlas 3. *NeuroImage*, 206(May 2019):116189, 2020. ISSN 10959572. doi: 10.1016/j.neuroimage.2019.116189.
