# Supplementary material for: Causal phase-dependent control of non-spatial attention in human prefrontal cortex
Source: Nat Hum Behav. 2024 Feb 16;8(4):743–57. doi: 10.1038/s41562-024-01820-z (PMC11045450; doi:10.1038/s41562-024-01820-z)
Supplement: Supplementary file 2 — Reporting Summary [file 41562_2024_1820_MOESM2_ESM.pdf]

nature portfolio

Corresponding author(s): Jeroen Brus, Joseph A. Heng, Rafael Polanía

Last updated by author(s): Dec 13, 2023

Reporting Summary

Nature Portfolio wishes to improve the reproducibility of the work that we publish. This form provides structure for consistency and transparency in reporting. For further information on Nature Portfolio policies, see our Editorial Policies and the Editorial Policy Checklist.

Statistics

For all statistical analyses, confirm that the following items are present in the figure legend, table legend, main text, or Methods section.

n/a	Confirmed	
☐	☒	The exact sample size (n) for each experimental group/condition, given as a discrete number and unit of measurement
☐	☒	A statement on whether measurements were taken from distinct samples or whether the same sample was measured repeatedly
☐	☒	The statistical test(s) used AND whether they are one- or two-sided<br>*Only common tests should be described solely by name; describe more complex techniques in the Methods section.*
☐	☒	A description of all covariates tested
☐	☒	A description of any assumptions or corrections, such as tests of normality and adjustment for multiple comparisons
☐	☒	A full description of the statistical parameters including central tendency (e.g. means) or other basic estimates (e.g. regression coefficient) AND variation (e.g. standard deviation) or associated estimates of uncertainty (e.g. confidence intervals)
☐	☒	For null hypothesis testing, the test statistic (e.g. F, t, r) with confidence intervals, effect sizes, degrees of freedom and P value noted<br>*Give P values as exact values whenever suitable.*
☐	☒	For Bayesian analysis, information on the choice of priors and Markov chain Monte Carlo settings
☐	☒	For hierarchical and complex designs, identification of the appropriate level for tests and full reporting of outcomes
☒	☐	Estimates of effect sizes (e.g. Cohen's d, Pearson's r), indicating how they were calculated

Our web collection on statistics for biologists contains articles on many of the points above.

Software and code

Policy information about availability of computer code

Data collection	Matlab R2019a, Psychtoolbox-3.0.14, SHINE toolbox
Data analysis	Matlab R2019a, R3.6.3, JAGS 4.3.0, Fsl v6.0.1&v5.0, FEAT v6.0.0, MCFLIRT, FLIRT, FNIRT, FILM, FLAME, Fieldtrip 2019.11.26, SPM12 and Sim4Life

For manuscripts utilizing custom algorithms or software that are central to the research but not yet described in published literature, software must be made available to editors and reviewers. We strongly encourage code deposition in a community repository (e.g. GitHub). See the Nature Portfolio guidelines for submitting code & software for further information.

Data

Policy information about availability of data

All manuscripts must include a data availability statement. This statement should provide the following information, where applicable:

- Accession codes, unique identifiers, or web links for publicly available datasets
- A description of any restrictions on data availability
- For clinical datasets or third party data, please ensure that the statement adheres to our policy

Source data for all figures can be found at the Open Science Foundation at: XX. Full data that support the findings of this study are available on request from the corresponding authors. The data are not publicly available due to information that could compromise the privacy of the participants. The study also made use of the Brainnetome Atlas https://atlas.brainnetome.org/ and the automated anatomical labelling atlas (AAL3v1) https://www.oxcns.org/aal3.html.

Human research participants

Policy information about studies involving human research participants and Sex and Gender in Research.

Reporting on sex and gender	This study tested 142 young volunteers of which 69 were male and 73 were female. Their sex was determined via self-reporting but was not relevant to any analysis we performeded.
Population characteristics	All participants were healthy and had normal or corrected to normal eye-sight. None of the participants suffered from any neurological or psychological disorder or took medication that interfered with participation in our study. In the fMRI experiment the mean age is 25.6, EEG 25.5, first tACS 25.8, second tACS 24.3 and third tACS 25.1.
Recruitment	Participants were recruited at the online participant recruitment platforms of the ETH Zürich and the UZH. This recruitment procedure biases towards young and highly educated participants, the impact of this recruitment bias on the results is hard to estimate.
Ethics oversight	Ethics Committee of the Canton of Zürich

Note that full information on the approval of the study protocol must also be provided in the manuscript.

Field-specific reporting

Please select the one below that is the best fit for your research. If you are not sure, read the appropriate sections before making your selection.

☐ Life sciences ☒ Behavioural & social sciences ☐ Ecological, evolutionary & environmental sciences

For a reference copy of the document with all sections, see nature.com/documents/nr-reporting-summary-flat.pdf

Behavioural & social sciences study design

All studies must disclose on these points even when the disclosure is negative.

Study description	We have acquired quantitative experimental data using fMRI, EEG and behavioral studies, including tACS.
Research sample	The study tested healthy young volunteers (n=142, age 18-40) recruited through the UZH and ETH web page available for recruitment of participants. The sample is representative of young healthy individuals studying at the ETH Zürich and the UZH. This follows common practices for participant recruitment at the ETH.
Sampling strategy	Sample size was determined based on previous studies using similar stimuli and tasks (Zanto 2011). Sampling procedure was based on voluntary response.
Data collection	The experiment was implemented in Matlab with the use of Psychtoolbox. Participants eye movements were recorded and depending on the experiment fMRI, EEG or tACS techniques were used. Nobody was present during the experiment except for the participant and experimenter. The reseacher was blinded to the stimulation condition.
Timing	Data was collected from March 2019 to September 2022.
Data exclusions	Four participants were excluded due to excessive noise in the EEG recordings. Participants with discrimination performance < 55% in block one (suggesting nearly random choice selection and therefore poor engagement) were excluded from the data analyses, resulting in the exclusion of 12 participants in experiments 3,4&5.
Non-participation	No participants dropped out or declined participation.
Randomization	Participants were not allocated to experimental groups.

Reporting for specific materials, systems and methods

We require information from authors about some types of materials, experimental systems and methods used in many studies. Here, indicate whether each material, system or method listed is relevant to your study. If you are not sure if a list item applies to your research, read the appropriate section before selecting a response.

Materials & experimental systems

n/a	Involved in the study
☒	☐ Antibodies
☒	☐ Eukaryotic cell lines
☒	☐ Palaeontology and archaeology
☒	☐ Animals and other organisms
☒	☐ Clinical data
☒	☐ Dual use research of concern

Methods

n/a	Involved in the study
☒	☐ ChIP-seq
☒	☐ Flow cytometry
☐	☒ MRI-based neuroimaging

Magnetic resonance imaging

Experimental design

Design type	Event related design
Design specifications	We tested 20 participants who each performed 192 trials divided over 6 blocks. Each trial took about 7 seconds with an intertrial interval between 1 and 3 seconds.
Behavioral performance measures	Correct button presses and reaction times were recorded. Performance over 55% was used as a criterion for sufficient task performance.

Acquisition

Imaging type(s)	functional and structural
Field strength	3 Tesla
Sequence & imaging parameters	Pulse sequence type was gradient echo with EPI imaging, FOV = 222.75x128, flip angle 85 degrees, slice angle of 20 degrees relative to the anterior-posterior commissure line.
Area of acquisition	Whole brain

Diffusion MRI ☐ Used ☒ Not used

Preprocessing

Preprocessing software	Analysis and pre-processing of the data was performed in FSL's Analsysis Tool FEAT v6.0.0, this included a BET brain extraction, slice timing correction, motion correction using MCFLIRT, a Gaussian spatial smoothing with a full width at half maximum of 5 mm, and a high pass temporal filtering with a cut-off of 100s
Normalization	Images were spatially normalized using FLIRT
Normalization template	The functional images were normalized to the high resolution structural image and then using FNIRT were warped onto the reference brain in MNI coordinate space.
Noise and artifact removal	There was a slice timing correction and a motion correcion using MCFLIRT.
Volume censoring	There was no volume censoring

Statistical modeling & inference

Model type and settings	First level analysis was performed with FILM based on general linear modelling with the canonical hemodynamic response function. Group-level analysis was performed using FMRIB's Local Analysis of Mixed Effects Tool.
Effect(s) tested	Contrasts were defined for attention to scene vs visual stimulus presentation and attention to motion vs visual stimulus presentation.

Specify type of analysis: ☒ Whole brain ☐ ROI-based ☐ Both

Statistic type for inference (See Eklund et al. 2016)	Cluster wise omparison with a cluster correction at a threshold of $P < 0.05$
Correction	-

Models & analysis

n/a	Involved in the study
☒	☐ Functional and/or effective connectivity
☒	☐ Graph analysis
☒	☐ Multivariate modeling or predictive analysis
